# Supplementary material for: Evaluation of suPAR as a Key Prognostic Biomarker in Patients with SARS-CoV-2 Pneumonia
Source: Biomedicines. 2025 Apr 8;13(4):896. doi: 10.3390/biomedicines13040896 (PMC12024740; doi:10.3390/biomedicines13040896)
Supplement: Supplementary file 1 [file biomedicines-13-00896-s001.zip › biomedicines-3557375-supplementary.pdf]

SUPLEMENTAL DATA

Supplementary Table S1. Biomarker values expressed as mean and interquartile range based on the studied outcomes.

	ICU-admission			In-hospital mortality		
	**YES (n=26)**	**NO (n=214)**	**p**	**YES (n=15)**	**NO (n=225)**	**p**
**SuPAR**	8.75 (6.25-12.00)	5.60 (3.98-8.40)	<0.001	12.20 (8.70-16.00)	5.70 (4.10-8.40)	<0.001
**Linfocitos**	825 (710-1465)	1170 (855-1468)	0.084	790 (460-1600)	1150 (820-1450)	0.187

Supplementary Table S2. Diagnostic performance based on Area Under the Curve (AUC) of the different biomarkers evaluated and the CURB-65 scale for the assessed outcomes.

	ICU-admission	In-hospital mortality
**SuPAR**	0.74 (0.66-0.82) p<0.0001	0.85 (0.76-0.94); p=0.045
**Lymphocytes**	0.60 (0.48-0.73); p=0.084	0.60 (0.41-0.80); p=0.187
**CURB65**	0.61 (0.49-0.73); p=0.077	0.90 (0.82-0.97); p=0.039
